# Clinical outcome of thymectomy in myasthenia gravis patients: A report from Iran

**Published:** 2018-01-05

**Authors:** Benyamin Seyfari, Farzad Fatehi, Abolfazl Shojaiefard, Mehdi Jafari, Ali Ghorbani-Abdehgah, Shirzad Nasiri, Aidin Yaghoobi-Notash, Behnam Molavi, Amir Hossein Latif, Reza Eslamian, Ali Mir, Ahmadreza Soroush

**Affiliations:** 1 Research Center for Improvement of Surgical Outcomes and Procedures, Shariati Hospital, Tehran University of Medical Sciences, Tehran, Iran; 2 Iranian Center of Neurological Research, Tehran University of Medical Sciences, Tehran, Iran; 3 Department of Surgery, School of Medicine, Tehran University of Medical Sciences, Tehran, Iran

**Keywords:** Thymectomy, Myasthenia Gravis, Clinical Outcome

## Abstract

**Background:** Myasthenia gravis (MG) is an autoimmune disease affecting acetylcholine postsynaptic receptor of voluntary muscles. Thymectomy is done in these patients and is a mainstay in the treatment of MG; however, the long-term result of surgery is still controversial. This study dealt with the investigation of the results of thymectomy in treatment, recovery and control of the symptoms of these patients.

**Methods:** This study was performed through a retrospective method in patients suffering from MG who underwent trans-sternal thymectomy between 2011 and 2016. We conducted thymectomy, excision of mediastinal mass and contents of tissues between the right and left phrenic nerves for all patients. Then, the effect of various variables including age, sex, time interval between onset of disease and surgery, thymus pathology and the dosage of drug on clinical response after surgery was determined using various statistical tests.

**Results:** 47 patients including 26 men and 21 women with the mean age of 33.0 ± 4.6 years have been investigated. The mean age of patients was 36.2 and 29.7 in men and women respectively (P = 0.041). Spiral chest computed tomography (CT) scan was present in 47 patients demonstrating mediastinal mass in 40 (85.1%) patients. Also, our pathological results showed thymic cells in aortopulmonary window contents of 4 patients. According to the results, the younger age of patients at the time of surgery, shorter time between diagnosis and thymectomy, being a woman and non-thymoma pathology were along with better clinical outcomes after thymectomy.

**Conclusion:** Our study shows better clinical results of thymectomy in patients with normal chest CT scan and normal or atrophic thymus in pathologic reports. Generally, it seems that performing thymectomy in a shorter time interval after diagnosis of MG is beneficial. Moreover, in MG patients who do not suffer from thymoma, it is along with positive results.

## Introduction

Myasthenia gravis (MG) is an autoimmune disease caused by antibodies probably originated from thymus glands. These antibodies attack acetylcholine receptors, resulting in voluntary skeletal muscle weakness. Thymectomy is done in these patients and is a mainstay in the treatment of MG.^1^^-^^3^ Rigorous physical activity amplifies this weakness.^4^ The physical symptoms of the disease affect the ocular muscles and can appear in mild, moderate, severe, or variable generalized forms (Osserman’s classifications). In the generalized form, hip and shoulder muscles are generally the first ones affected. In more severe forms, weakness of the bulbar muscles can cause dysphasia and dysarthria. These symptoms are characterized by a marked increase as a result of exercise. In severe and untreated forms of the illness, the patient experiences respiratory muscle weakness and respiratory failure. 

MG is diagnosed by the patient’s history, physical symptoms, paraclinical tests such as the level of antibodies against the acetylcholine receptors and slow-RNS (3-H2 repetitive nerve stimulation). The relationship between MG and the thymus gland is not exactly clear, but it has been observed that most patients with MG display various degrees of thymus abnormality.^5^^,^^6^ Thymectomy is performed when there is resistance to the drug regimen or the patient is deemed unfit for drug therapy. In patients with generalized MG (GMG) or patients who have MG with a tumor in the mediastinum, thymectomy is widely used.^7^ During thymectomy, the thymus gland, the tumor and its adjacent tissue, and the tissue between the right and left phrenic nerves are removed.^8^^,^^9^

Sauerbruch tried thymectomy for treating MG in 1911 for the first time. The modern procedure for thymectomy was started by Alfred Blalock at John Hopkins Hospital in 1936.^10^ In patients with MG and a history of myasthenia crisis, thymectomy leads to favourable outcomes.^2^ Thymectomy can also reduce the mortality and increase recovery.^11^^,^^12^ Antibodies against acetylcholine receptors are found in a significant portion of MG patients; therefore, drug therapy using substances that preserve acetylcholine is the first step in treating this disorder. These drugs include acetylcholine’s inhibitors (AchEI) at the early stages, and immunosuppressant agents such as corticosteroids and Imuran at the later stages.^12^^,^^13^ If a patient cannot tolerate the drugs, or a tumor is found within the mediastinum or the thymus, or the disorders do not respond to drug therapy, or myasthenia crisis is observed, thymectomy will be performed after immunoglobulins (Ig) are prescribed or plasmapheresis is performed and the patient is deemed stable.^14^^-^^16^ The main reason for this operation is the high percentage of thymus abnormalities in MG patients. Thymectomy can be performed at any age for treating MG and taking out the entirety of the thymus tissue increase the chance of recovery.^12^ Practically, comparing thymectomy and drug therapies is not possible due to their different indications; however, some studies have attempted to do this.^9^ This study was performed to evaluate the results of thymectomy for MG patients.

## Materials and Methods

A retrospective study was performed on 47 individuals diagnosed with MG who underwent thymectomy in Shariati hospital, Tehran, Iran, during 2011-2015. MG was diagnosed using the patient’s history, physical examination, laboratory tests and electromyography. The level of antibodies against acetylcholine receptors was the main lab test used in this study. The presence of these antibodies is consistent with MG diagnosis. Electro-physiologic tests can also be useful. In GMG the RNS test is positive in 85.0% of the cases. When uncertainty exists, the single fiber electromyography (SFEMG) test is used. If the results of SFEMG are negative then MG dose not exist. Thymectomy was performed when radiographic [computed tomography (CT), and chest X-ray (CX-ray)] revealed tumors within the mediastinum or the thymus. Thymectomy was also performed in cases when drug therapy was not possible (patient did not respond well to drug therapy, or the patient was deemed not fit enough for drug therapy) or myasthenia crisis. In patients with myasthenia crisis, the operation was performed after they were stabilized using plasmapheresis, immunoglubin, and other medications. 

During thymectomy, a partial or total sternotomy was performed by just one surgeon. Afterwards, the thymus, the mediastinal tumor (if existed), the surrounding tissues, and the tissues between the right and left phrenic nerves were removed. The inclusion criteria were patients who suffered from MG and underwent thymectomy with range age of 10-60 years. Follow-up CT scans were performed after one year for all patients. 

The follow-up criteria were physical symptoms and need for medication. Patients who did not need any medication at the one-year mark were deemed fully recovered. A reduction in the medication dosage was the criterion for partial recovery. 

**Table 1:** Clinical characteristic at baseline and clinical improvement of patients

**Variable**	**Level**	**n (%)**
Osserman’s classification	Eye (I)	2 (4.2)
	Mild generalized disease (IIA)	11 (23.4)
	Disease moderate severity generalized (IIB)	21 (44.7)
	Acute and progressive disease (III)	4 (8.5)
	Severe disease and late (IV)	9 (19.1)
Neurological findings at baseline	Bulbar	2 (4.2)
	Bulbar + ocular	4 (8.5)
	Bulbar + muscular weakness	6 (12.8)
	Ocular	14 (29.8)
	Ocular + muscular weakness.	5 (10.6)
	Muscular weakness	6 (12.8)
	Bulbar + muscular weakness + ocular	10 (21.3)
Clinical improvement	Complete improvement	16 (34.0)
	Relative improvement	24 (51.0)
	Without clinical changes	1 (2.1)
	Intensified	6 (12.7)

Patients without a reduction or increment in medication dosage were put in the no-recovery group.^9^ The results were analyzed using various statistical tests. At the beginning, normality of variables was analyzed by Kolmogorov-Smirnov test, then the data were compared using statistical tests such as Student's independent t-test, Mann-Whitney, chi-square and Fisher’s exact tests. SPSS software (version 22, IBM Corporation, Armonk, NY, USA) was used for analysis. Statistical significance was defined as confidence interval (CI) > 95% and P < 0.050.

## Results

47 patients (26 men and 21 women) with the mean age of 33.0 ± 4.6 were studied. The mean age of man participants was higher (36.2 in men vs. 29.7 years in women) (P = 0.041). Ophthalmologic symptoms and muscle weakness were the most common neurologic findings at the beginning of the sickness and during its course (Table 1). 72.0% of the patients in this study were found to be in class II category, according to Osserman’s system (Table 1). Mediastinal CT scan was performed on all patients. In 40 patients (85.1%), a tumor was found within the mediastinum or the thymus. No tumors were found in seven patients (26.0%) and they were reported as normal. 

In 22 patients (46.8%), surgery was performed less than a year after the onset of MG. 20 Patients (42.5%) underwent the operation between 1-3 years after onset. In 5 (10.6%) cases, the surgery was done more than three years after the onset. 

Results showed that patients were generally satisfied with the performed surgery and their recovery was improved. Full recovery and partial recovery was achieved in 16 (34.0%) and 24 (51.0%) patients, respectively, while in 7 patients (14.9%) there was not any improvement in their condition. Moreover, based on the follow-up results, one patient died and complications were found in 2 cases (phrenic nerve palsy and sternomediastiniis). Furthermore, in patients with the shorter gap between the onset of symptoms and operation, the recovery was improved (Table 2). In addition, in patients who had no thymoma in pathologic samples, the recovery was improved.

**Table 2:** Effective factors on clinical outcomes in patients with myasthenia gravis

**Variable**		**Improved**		**Not improved ** **(n = 7)**	**P** ^*^
		**Complete improvement ** **(n = 16 )**	**Relative improvement ** **(n = 24)**		
Age (year) (mean ± SD)		25.4 ± 17.5	27.9 ± 18.4	34.6 ± 12.2	0.038
Sex [n (%)]	Woman	7 (43.7)	11 (45.8)	3 (42.9)	0.045
	Man	9 (56.3)	13 (54.2)	4 (57.1)	
Gap between onset of symptoms and operation (year) (mean ± SD)		0.8 ± 0.3	1.5 ± 0.2	2.8 ± 1.6	0.022
Thymoma [n (%)]		10 (62.5)	15 (62.5)	6 (85.7)	0.040

SD: Standard deviation

* Student's independent t-test or Mann-Whitney test or chi-square test or Fisher’s exact test

## Discussion

This study showed evidence supporting the use of trans-sternal thymectomy for improving partial and complete recovery of MG patients and reducing the need for drug therapy in these patients. In this study, physical symptoms, serologic and electrophysiological tests were used. Patients were classified using Osserman’s system and most patients were diagnosed with the mild and moderate GMG. All MG patients underwent drug therapy at first and then patients who had mediastinal mass or did not respond to drug therapy underwent thymectomy. Most findings show that thymectomy is effective in treating these patients. Wolfe, et al. in a randomized clinical trial provided evidence about the effect of thymectomy for improving clinical outcomes in myasthenia patients.^12^ Moreover, Hatton, et al. emphasized the positive effects of thymectomy on moderate and advanced MG.^17^ Gronseth and Barohn found that thymectomy was more effective in the first stage of the sickness, and surgeries performed during the later stages of the sickness had less effect.^18^ This may be due to the irreversible destruction of acetylcholine receptors. 

The current study also found that earlier operations resulted in better recovery. Other factors correlated with better prognosis were ages of less than 30 years, non-thymoma pathology, and being a woman.^19^ These findings were supported by other studies.^20^ Median sternotomy prepares a wide exploration area and it is preferred by many chest surgeons and neurologists on the grounds. It provides a higher amount of thymic tissue and limited risk of phrenic nerve injuries.^1^^,^^9^ Our patients also underwent median sternotomy, thymectomy, tumor excision and remain tissues between both right and left phrenic nerves.

Beers and Berkow^14^ found that thymoma was associated with worse prognosis which is consistent with our study. Multiple studies have reported full recovery rates of 30%-55% after thymectomy.^2^^,^^9^^,^^10^ Whereas, 34% of the patients in the current study fully recovered after surgery and about half of them (51%) experienced partial recovery during the follow-up period. It means over 85% of studied patients have experienced total or partial recovery. 

Our hospital is an academic referral center and mainly we see patients with thymic tumors. Since the patients do not respond well to medical management, the early referral could potentially improve our outcomes. One of our patients who received prednisolone and had a history of diabetes mellitus involved with sternomediastinitis. Based on these findings in patients with MG who have treated with high dose prednisolone and have a history of diabetes mellitus, tapering of prednisolone before surgery should be considered. 

The main limitation of this study was the low sample size, which could affect the accuracy of the statistical comparisons. The results of this study suggest that thymectomy can reduce patient’s need for medication and reduce the severity of MG regardless of age, sex, severity or length of sickness, or thymoma.

## Conclusion

This study shows the better physical results of thymectomy in patients with MG displaying normal mediastinum CT scans with normal or atrophied thymuses. Factors correlated with improved physical symptoms after thymectomy were lower patient age, being a woman, less time between onset of symptoms and surgery, and pathology without thymoma. Overall, thymectomy immediately after the diagnosis of MG is suggested to be beneficial.

## Notes:

**How to cite this article:** Seyfari B, Fatehi F, Shojaiefard A, Jafari M, Ghorbani-Abdehgah A, Nasiri S, et al. Clinical outcome of thymectomy in myasthenia gravis patients: A report from Iran. Iran J Neurol 2018; 17(1): 1-5.
